# Role and regulation of heme iron acquisition in gram-negative pathogens

**DOI:** 10.3389/fcimb.2013.00055

**Published:** 2013-10-08

**Authors:** Laura J. Runyen-Janecky

**Affiliations:** Department of Biology, University of Richmond, RichmondVA, USA

**Keywords:** heme, hemin, hem, hemoglobin, iron, pathogens, regulation, Fur

## Abstract

Bacteria that reside in animal tissues and/or cells must acquire iron from their host. However, almost all of the host iron is sequestered in iron-containing compounds and proteins, the majority of which is found within heme molecules. Thus, likely iron sources for bacterial pathogens (and non-pathogenic symbionts) are free heme and heme-containing proteins. Furthermore, the cellular location of the bacterial within the host (intra or extracellular) influences the amount and nature of the iron containing compounds available for transport. The low level of free iron in the host, coupled with the presence of numerous different heme sources, has resulted in a wide range of high-affinity iron acquisition strategies within bacteria. However, since excess iron and heme are toxic to bacteria, expression of these acquisition systems is highly regulated. Precise expression in the correct host environment at the appropriate times enables heme iron acquisitions systems to contribute to the growth of bacterial pathogens within the host. This mini-review will highlight some of the recent findings in these areas for gram-negative pathogens.

## Introduction

Almost all living organisms require iron for growth. One notable exception is the Lyme disease pathogen, *Borrelia burgdorferi*, which uses manganese in place of iron (Posey and Gherardini, 2000). Iron is critical for a wide range of cellular functions; however, high levels of iron are toxic because iron catalyzes the formation of reactive oxygen species, and iron acquisition by cells is highly regulated as a result. In the complex interaction between human host and bacterium, iron plays a critical role. Free ferric (Fe^3+^) iron is poorly soluble in aerobic conditions at neutral pHs; however, ferrous (Fe^2+^) iron is much more soluble. Additionally, the host sequesters free iron in iron binding proteins (such as ferritin, transferrin, lactoferrin) and in heme and hemoproteins to prevent iron toxicity and to withhold nutrients from pathogens, thereby limiting pathogen growth. Thus, free iron is not readily available to the bacterial pathogen inside the host. Pathogens have evolved numerous mechanisms to capture this limited supply of free iron and iron from host iron proteins. Since the type of iron available varies depending on the location of the pathogen within the human host and since pathogens occupy a wide variety of host niches, there is a diversity of iron acquisition mechanisms employed by both intracellular and extracellular pathogens. This mini-review focuses on acquisition of iron in gram-negative pathogens from one of the most abundant sources—host heme.

## Availability of heme and heme-containing molecules in the human host

Approximately 70% of the iron in the human body is within heme, a heterocyclic organic ring called porphryin covalently bound to one ferrous iron atom (Bridges and Seligman, 1995). Heme is critical for functions including oxygen transport, enzymatic reactions, and cellular respiration. Heme is synthesized in almost all human cell types (the majority in erythroid cells, and to a lesser extent in hepatocytes) and can be obtained from the diet (reviewed in Hamza and Dailey, 2012).

Heme is an essential biomolecule; however, excess free heme is toxic to cells due to its lipophilic nature, lipid peroxidation capacity, and ability to catalyze the production of reactive oxygen species (reviewed in Anzaldi and Skaar, 2010). Thus, over 95% of the heme is bound to proteins (hemoproteins), the majority of which are intracellular (Bridges and Seligman, 1995). The intracellular free heme pool is approximately 0.1 μM, which is less than 0.1% of total cellular heme (Granick et al., 1975). The majority of heme in the human body (~67%) is in hemoglobin, which is primarily found in erythrocytes (Bridges and Seligman, 1995). Other major hemoproteins include myoglobin and cytochromes. Recently, additional hemoproteins have been described, including cytoglobin and neuroglobin, which appear to play a role in oxygen homeostasis/oxygen stress (Liu et al., 2012b; Watanabe et al., 2012; Storz et al., 2013). Additional heme binding proteins exist that are most likely important in scaffolds for synthesis and scavenging heme. The existence of heme chaperones for incorporating heme into apo-hemoproteins has been proposed, but such proteins have yet to been identified in humans (Severance and Hamza, 2009). All of these proteins represent potential heme sources for intracellular pathogens.

Although the majority is intracellular, limited amounts of heme can be found extracellular and thus available to extracellular pathogens. One of the major locations for extracellular heme is in blood hemoglobin (estimated to be 80–800 nM in serum) (Schryvers and Stojiljkovic, 1999). Hemoglobin from lysed erythrocytes is bound by haptoglobin for eventual recycling by macrophage and hepatocytes (Tolosano et al., 2010). Free heme, from damaged hemoglobin, is bound by serum hemopexin and, to a lesser extent, serum albumin. In the gut, dietary heme may be bioavailable to bacteria, either free or complexed with hemopexin. Heme levels are thought to be low in the respiratory track; however, since the heme auxotroph *Haemophilus influenzae* can live in this environment, there must be enough heme to support bacterial growth (Fournier et al., 2011). The urogenital track has varying amounts of heme: the bladder, urethra, and male genital track likely have low heme levels; however, there may be high heme levels in the female urogenital track during menses (Schryvers and Stojiljkovic, 1999). Finally, even in environments where heme is typically low, heme and hemoproteins are released by cells damaged during infection.

## Bacterial heme transporters and liberation of iron from heme

Host microenvironments that have potential heme sources have selected for bacteria with high-affinity heme transport systems which locate and transport heme into the bacterial cell. Heme auxotrophs can use the intact heme for insertion into bacterial hemoproteins. Additionally for both heme prototrophs and autotrophs alike, the iron can be extracted from the heme for other uses (e.g., building Fe-S cluster proteins). Most commonly, there is direct uptake of heme by a cell surface receptor which binds heme or host hemoproteins. A variation of this method includes bipartite systems in which a lipoprotein facilitates heme or hemoproteins binding to the cell surface receptor (Lewis et al., 1998, 2006). Alternatively, some pathogens produce hemophores, small secreted proteins that capture free heme or heme bound to host hemoproteins and then deliver this heme to bacterial surface receptors (Cescau et al., 2007).

**Figure 1:**
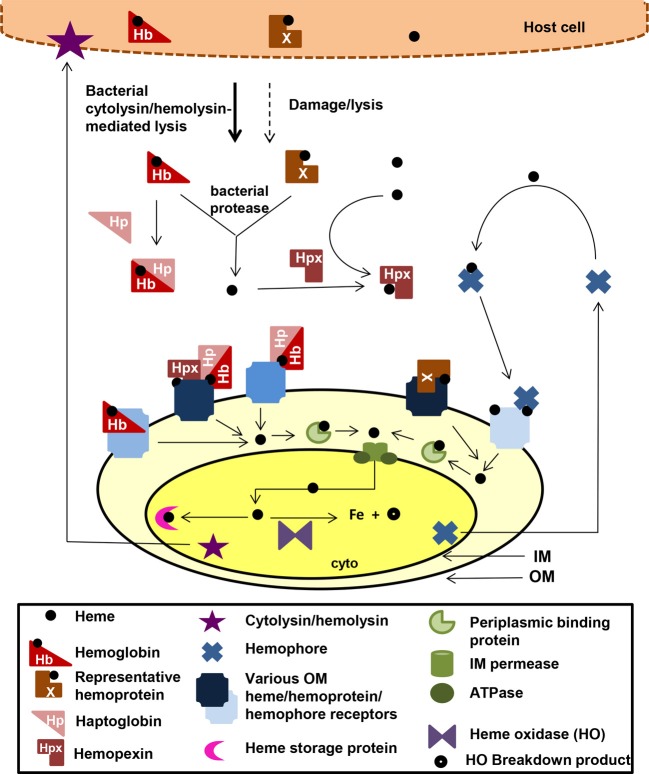
**Mechanisms for heme iron acquisition from the host by gram-negative bacteria.** Bacteria factors damage host cells releasing heme, Hb, and other hemoproteins. Additionally, secreted bacterial hemophores capture host heme. Extracellular host Hb and heme may be bound by host Hp and Hpx, respectively. A bacterium could acquire iron from these host heme sources using one or more TonB-dependent outer membrane (OM) receptors for these heme compounds, which transport the heme through the outer membrane into the periplasm. Some OM receptors are specific for one molecule, whereas others have a broad specificity for multiple hemoproteins. Transport though the periplasmic and across the inner membrane is facilitated by ABC transport systems (green). Inside the bacterium, the heme is degraded using heme oxidases or stored in heme storage protein. Intracellular pathogens would have access to host heme and hemoproteins via similar mechanisms. cyto, bacterial cytoplasm; IM, bacterial inner/cytoplasmic membrane; OM, bacterial outer membrane. Although all the OMR are TonB-dependent, TonB is not shown in the figure.

There are over 30 well-characterized outer membrane heme receptors that transport heme in gram-negative pathogens, although there are many more putative receptors in genomic databases (Table 1). The overall structure of these proteins includes a membrane spanning beta-barrel with extracellular loops that bind to free heme, host hemoproteins, or bacterial hemophores (reviewed in Wilks and Burkhard, 2007). Most are characterized by the presence of FRAP/NPNL domains with a conserved histidine residue that coordinates that heme (Stojiljkovic et al., 1995), although there are reports of heme transporters lacking some of these elements (e.g., PhuR from *Pseudomonas aeruginosa*) suggesting that there are other motifs for heme coordination in outer membrane heme transporters (Tong and Guo, 2009). The energy for heme transport is transduced from the inner to the outer membrane using the TonB/ExbB/ExbD system (reviewed in Krewulak and Vogel, 2011). Thus, all heme outer membrane transporters have a characteristic “TonB box” motif, through which the receptor interacts with TonB. Given the presence of multiple hemoproteins as potential iron sources, there are at least two strategies for bacteria to optimize access to heme iron (Figure 1). Some species have multiple receptors, presumably for different hemoproteins or for expression in different host environments (e.g., *Haemophilus influenza*). Other species have one outer membrane receptor capable of binding to multiple hemoproteins (e.g., *Yersinia enterocolitica* HemR), suggesting the recognition is at the level of the heme molecule (Stojiljkovic and Hantke, 1992; Bracken et al., 1999).

**Table 1:**
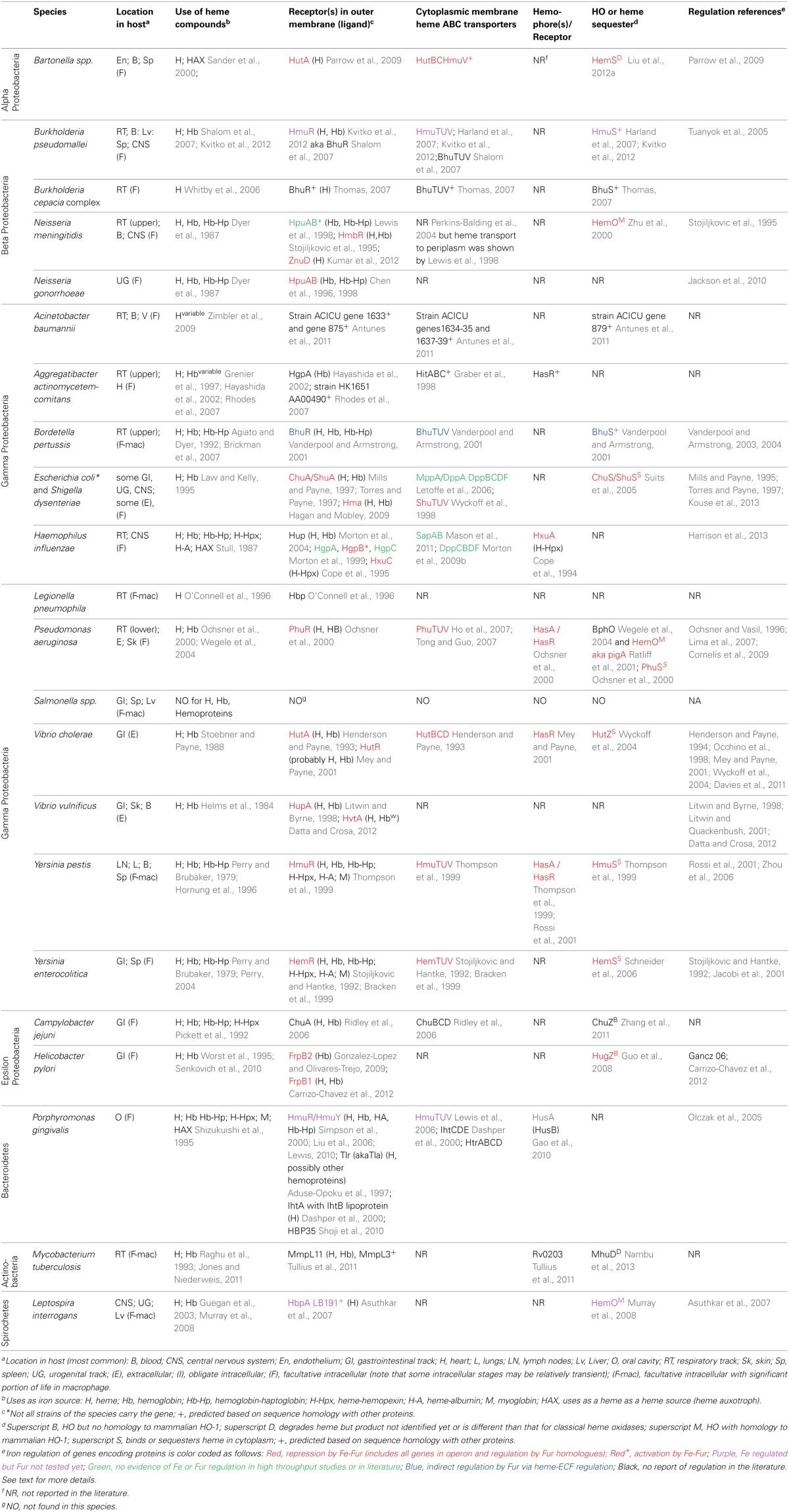
**Characteristics of heme iron acquisition in some major pathogens**.

Once the heme molecule has been transported through the outer membrane receptor, ABC transport systems then transport heme though the periplasm, across the inner membrane, and into the cytoplasm (Table 1 and Figure 1). Each ABC transport system consists of a high-affinity periplasmic ligand-binding protein which shuttles heme through the periplasm, two subunits of a cytoplasmic membrane permease, and a peripheral membrane ATPase that supplies the energy for transport. Although there is low sequence homology among the approximately 50 identified periplasmic heme binding proteins, all but one has a conserved tyrosine which is believed to coordinate heme (Tong and Guo, 2009). Frequently, these ABC transporter genes are located in the same operon as or near outer membrane receptor genes; however, orphan ABC transporters that can transport heme exist (e.g., the *E. coli* DppABCD system, which also transports dipeptides) (Letoffe et al., 2006).

Upon entry into the bacterial cell, heme storage, transfer and degradation proteins sequester heme and facilitate extraction of the iron from heme (Table 1 and Figure 1). Bacterial proteins that sequester heme likely prevent heme from catalyzing the formation of reactive oxygen species [e.g., *Shigella dysenteriae* ShuS Wyckoff et al. (2005)]. Other cytoplasmic heme-binding proteins transfer heme to heme degradation proteins [e.g., *Pseudomonas aeruginosa* PhuS Lansky et al. (2006)]. Many pathogens contain homologues of mammalian heme oxygenases (HO), enzymes that cleave the heme to release the iron, generating biliverdin and CO as end products (e.g., *Pseudomonas aeruginosa* HO and *Neisseria meningitidis* HemO). Recently, new structural classes of HOs have been identified such as the “split-barrel fold class” in *Helicobacter pylori* (HugZ) and *Campylobacter jejuni* (ChuZ) (Guo et al., 2008; Zhang et al., 2011). Additional bacterial enzymes that degrade heme to liberate iron, but release different end products than those released by classical HOs, have been identified. For example, MhuD in *Mycobacterium tuberculosis* cleaves heme to release the iron, generating a novel tetrapyrrole product of called mycobilin, but not CO (Nambu et al., 2013).

For pathogens that can transport heme, the ability to increase the local concentration of heme and/or hemoproteins would be advantageous for growth in the host. Production of cytolysins/hemolysins that lyse cells releasing hemoproteins is common in almost all extracellular and facultative intracellular pathogens that use heme as an iron source. Additionally, some pathogens secrete proteases that degrade hemoproteins to release heme. For example, *Porphyromonas gingivalis* produces hemolysins to lyse cells and proteases called gingipans that have hemaglutin domains and degrade hemoproteins (Chu et al., 1991; Sroka et al., 2001). Alternatively, some bacteria secrete hemophores, small, secreted proteins that capture free heme or heme bound to host hemoproteins and that deliver the heme to bacterial cells. There are several distinct families of hemophores, which share little to no sequence similarity, suggesting convergent evolution of this strategy for increasing local heme concentration (Table 1; Figure 1). The first class of hemophores identified was the HasA group, initially characterized in *Serratia marcescens* (Letoffe et al., 1994). HasA captures heme, using conserved His32 and Tyr75 residues, and relays it to the outer membrane receptor HasR for transport. Homologues of the HasA/HasR system have only been found in gram-negative bacteria including *Yersinia pestis* (Rossi et al., 2001) and *Pseudomonas aeruginosa* (Ochsner et al., 2000). A second type of hemophore, only found in *Haemophilus influenza*, is HxuA, which captures heme from hemopexin, and the released heme is transported into the cell by outer membrane heme transporters (Fournier et al., 2011).

## Regulation of expression of heme iron acquisition genes by iron, heme and other stimuli

Most genes encoding components of heme iron acquisition systems are not transcribed in iron-replete condition because (1) high-affinity heme iron acquisition is generally not needed and, thus, would be energetically wasteful and (2) excess iron is cytotoxic. One of the most common mechanisms of regulation of heme iron acquisition system expression by iron levels utilizes iron-responsive transcriptional regulators that repress transcription of high-affinity iron acquisition systems when iron is plentiful. The prototypical example is Fur (ferric uptake regulation). In the classical model of iron-repression, Fe-Fur binds to a DNA sequence called the Fur-box in promoters of many high-affinity iron acquisition genes. Fe-Fur occupation of the promoter prevents RNA polymerase binding, thereby repressing transcription. When iron levels decrease, the Fe-Fur equilibrium shifts, Apo-Fur cannot bind to the Fur-box, and transcription occurs [for review Carpenter et al. (2009)]. DtxR and IdeR are iron responsive regulators with similar functions to Fur, and most heme acquisition genes are regulated by repressor proteins from the Fur or DtxR families (Table 1).

Not only is excess iron toxic to bacteria, but heme can also be cytotoxic due to its ability to catalyze the formation of reactive oxygen species, its peroxidase activity, and its lipophilic nature which disrupts cell membranes. Thus, for these reasons and for energetic reasons similar to those for iron regulation, expression of a subset of heme iron acquisition systems is regulated by heme levels in some pathogens. In *Bartonella quintana*, transcription of the *hut* operon increases when heme concentrations are lower than required for optimal growth, but decreases at very high heme concentrations. The decrease in expression is predicted to be mediated by the heme-responsive Irr transcriptional regulator, which is only found in some alpha-proteobacteria (Parrow et al., 2009). *Bordetella pertussis* employs an extracytoplasmic function σ factor (ECF) called HurI and its cognate anti-sigma factor HurR to modulate transcription of the *bhuRSTUV* heme uptake operon by heme though a mechanisms in which iron regulation and heme regulation converge. In low iron, Fur repression of *hurIR* is relieved; however, HurI is inactive because it is bound by HurR when heme is absent. Heme binding by BhuB alleviates HurR repression of HurI activity, and HurI can activate transcription of the *bhuRSTUV* operon. (Vanderpool and Armstrong, 2003, 2004). In the presence of heme, the *Vibrio vulnificus* LysR-family transcriptional regulator HupR increases transcription of the Fur-regulated outer membrane heme receptor gene *hupA* (Litwin and Quackenbush, 2001). In *Pseudomonas aeruginosa*, transcription of the *phu* operon is up-regulated via an uncharacterized, but Fur-independent, mechanism (Kaur et al., 2009). Regulatory patterns like these enable expression of heme iron acquisition systems when some heme is available for transport and/or prevent expression of the systems when heme levels are too high. It is unclear why more heme iron acquisition systems are not under such control; however, most expression studies have not formally tested this possibility and, thus, this mode of regulation may be more widespread than reported.

In addition to heme/iron levels, other host-related environmental stimuli may fine-tune expression of heme iron acquisition genes, allowing integration of the iron/heme conditions with other physiological and environmental signals. The cyclic AMP receptor protein, which actives transcription when glucose levels are low, activates expression of *Vibrio vulnificus hupA* (Oh et al., 2009). In *Shigella dysenteriae* and pathogenic *E. coli*, expression of the Fur-regulated outer membrane heme receptor genes *shuA* and *chuA* increases at 37°C due to post-transcriptional regulation by the 5' untranslated region of these genes (Kouse et al., 2013). The Fur-regulated *Yersinia pestis hasRADEB* and *Vibrio vulnificus hupA* genes have increased expression at 37°C and 40°C, respectively, as compared to lower temperatures (Rossi et al., 2001; Oh et al., 2009). *phuR* and *hasA* expression in *Pseudomonas aeruginosa* and *hmuRY* expression in *Porphyromonas gingivalis* are quorum/cell density-regulated (Arevalo-Ferro et al., 2003; Wu et al., 2009). *Haemophilus influenzae* and *Neisseria meningitidis* overlay phase variation on expression of heme acquisition systems, perhaps to counteract the host response to immunogenic OMPs (Ren et al., 1999; Richardson and Stojiljkovic, 1999). Finally, the pathogen's niche may change during the course of infection due to the interaction between host and pathogen and the movement of the pathogen through the host, and available iron sources may change as a result. Tissue specific expression of heme receptors has been show in several pathogens including *Yersinia enterocolitica*, where *hemR* expression is higher in spleen and peritoneum, as compared to liver and intestinal lumen. Furthermore, peritoneum expression of *hemR* is higher than in *in vitro* iron-limited media suggesting there are additional host specific signals besides low iron that allow for maximal *hemR* expression (Jacobi et al., 2001). Finally, there are examples of transcriptional regulation by other regulators suggesting there are more regulatory signals and integration with other regulatory pathways to be discovered.

In summary, each pathogen fine-tunes expression of heme iron acquisition genes to generate the appropriate physiological response for each environmental niche. This response is characterized by particular host heme iron sources/levels, total iron levels, other environmental inputs, and the phylogenetic history of the pathogen. Thus, there are varying patterns of regulation of heme iron acquisition system and regulation of the expression of these systems sometimes overlaps with other global regulatory circuits, creating intricate regulatory pathways in some pathogens. Alternatively, regulation of heme acquisition systems in other pathogens may be relatively simple (e.g., only regulated by an iron-responsive transcriptional regulator) because the pathogen is in a stable environment with low free iron and access to heme.

## Conclusions and future outlook

Although much is known about heme transport mechanisms and their regulation in many of well-studied pathogens, these topics have not been investigated as extensively in less-common and emerging pathogens, leaving the potential for novel discoveries. Furthermore, the possible fates of the transported heme molecule within the bacterial cell are just beginning to be clarified fully. Additional families of heme iron acquisition and utilization proteins may be waiting to be identified using biochemical (e.g., heme binding assays), genetic (e.g., complementation of *E. coli* heme mutants), and bioinformatic (e.g., mining expression databases for Fur- or iron-regulated genes and searching for heme binding motifs in proteins databases) approaches. Defining the role of each particular heme iron acquisition system in virulence is ongoing for many pathogens, but has been complicated by the presence of redundant systems in some pathogens and/or the use of certain systems in just one niche in the host. Thus, deletions of particular heme iron acquisition genes do not always show an effect in all animal models. It is clear, however that in many pathogens there is a role for some heme iron acquisition proteins, demonstrating the importance of heme for pathogenesis (Henderson and Payne, 1994; Morton et al., 2004, 2007, 2009a; Palyada et al., 2004; Domenech et al., 2005; Brickman et al., 2006; Hagan and Mobley, 2009). A more complete description of heme acquisition and utilization in human pathogens may serve as a reference point for understanding iron acquisition in non-pathogenic symbiotic bacteria that reside in humans and other animals, an area that is currently under-investigated. With respect to gene regulation, expression of the genes encoding most heme iron acquisition systems increases when iron is low due to alleviation of transcriptional repression by iron-responsive transcriptional repressors. However, whether heme levels and/or other regulatory RNAs or proteins modulate this expression further has not been examined for many of these genes.

Pathogens and their human hosts have evolved together, and as a consequence, there is a complex interplay between sequestration of iron from the pathogen by the host and elaboration of mechanism to capture that iron by the pathogen. From the host side, human hemoglobin is quite variable in amino acid sequence; thus, individuals may have differing susceptibility to pathogens due to differences in the ability of the pathogen to bind hemoglobin to access the heme (Pishchany and Skaar, 2012). Thus, bacteria pathogen acquisition of heme iron could have been a driving force for hemoglobin evolution. From the pathogen side, the fact that most heme is intracellular and bound to hemoproteins may have been a selective pressure for intracellular growth and protease/hemolysin production in pathogen evolution. Furthermore, heme acquisition genes have been found associated with mobile genetic elements in some pathogens (e.g., *Neisseria meningitidis* and *Shigella dysenteriae*), suggesting potential for rapid spread of these genes via horizontal gene transfer (Wyckoff et al., 1998; Kahler et al., 2001).

## Author contributions

Laura Runyen-Janecky conceived and wrote the entire manuscript.

### Conflict of interest statement

The author declares that the research was conducted in the absence of any commercial or financial relationships that could be construed as a potential conflict of interest.
